# CD40 agonism enhances immune checkpoint blockade and generates immunologic memory via CD4^+^ T cells in ERα+ mammary tumors

**DOI:** 10.21203/rs.3.rs-6823527/v1

**Published:** 2025-06-16

**Authors:** Casey Lam, Olivia Lanchoney, Vishnu Maddipatla, Nune Markosyan, Nikhil Joshi, Courtney Ray Fofana, Shan Zeng, Ronald P. DeMatteo, Robert H. Vonderheide, Jennifer Q. Zhang

**Affiliations:** University of Pennsylvania; University of Pennsylvania; University of Pennsylvania; University of Pennsylvania; University of Pennsylvania; University of Pennsylvania; University of Pennsylvania; University of Pennsylvania; University of Pennsylvania; University of Pennsylvania

## Abstract

There has been marked improvement in the clinical outcome of triple-negative breast cancer (TNBC) with the use of immune checkpoint blockade (ICB) although serious immune-related adverse effects are not uncommon. Unlike TNBC, ERα + breast tumors are largely unresponsive to ICB. Here we demonstrate defective priming by cross-presenting conventional dendritic cells (cDCs) and a blunted response to ICB in ERα + mouse mammary tumors compared to TNBC. Systemic administration of an agonistic CD40 antibody (aCD40) induced T cell proliferation and activation in tumor-draining lymph nodes and attracted effector T cells to the tumor bed from the periphery. This effect was largely due to activation, maturation and migration of type 1 conventional dendritic cells (cDC1s). aCD40 alone slowed tumor growth in ERα + tumors but its combination with ICB cured tumor-bearing mice, accomplishing a “vaccine effect” and the immune-mediated rejection of tumor rechallenge. The anti-tumor effect of aCD40 effect was cDC1 and CD8 + T cell-dependent, whereas the rejection of secondary tumor rechallenge in cured mice required CD4 + T cells. Importantly, intra-tumoral administration of aCD40 combined with systemic or intra-tumoral ICB – to mimic neoadjuvant therapeutic approaches—induced complete regressions of both treated and distant tumors. These findings indicate that aCD40 achieves DC activation required for the response to immunotherapy in ERα + tumors and further supports intra-tumoral administration of both aCD40 and ICB as an effective treatment that might limit systemic exposure and lower risk of immune-related toxicity.

## Introduction

Breast cancer remains a leading cause of cancer-related mortality in women worldwide, with estrogen receptor positive (ER+) subtypes accounting for approximately 70% of all cases^1,2^. Although many patients have a good prognosis, a significant proportion of patients will have locally advanced disease with a high risk of distant disease and death^3^ or develop endocrine therapy resistance^4,5^. Unfortunately, traditional chemotherapy has poor efficacy in ER + subtypes with low rates of complete pathologic response^6^, highlighting the urgent need for alternative therapeutic approaches. Immune checkpoint blockade (ICB), which has revolutionized treatment paradigms across multiple cancer types, has demonstrated only modest efficacy in breast cancer, with anti-PD-1/PD-L1 and anti-CTLA4 monoclonal antibodies benefiting a small subset of patients with predominantly PD-L1-positive tumors^7–10^. ICB in ER + breast cancer has shown largely limited efficacy^11,12^, primarily attributed to an immunologically “cold” tumor microenvironment (TME) including low density of tumor-infiltrating lymphocytes (TILs) and low tumor mutational burden^13–15^. Conversely, checkpoint blockade has demonstrated clinical efficacy in triple-negative breast cancer (TNBC)^16–18^, likely due to having a more activated tumor immune microenvironment compared to ER + disease. We thus pursued an alternative strategy to convert immunologically cold ER + breast cancers into more highly inflammatory tumors capable of improved responsiveness to checkpoint blockade.

CD40, a member of the tumor necrosis factor receptor superfamily expressed predominantly on antigen-presenting cells (APCs)^19^, represents a promising immunotherapeutic target^20^. Activation of CD40 through agonistic antibodies initiates a cascade of immunological events critical for effective anti-tumor immunity^21^. Specifically, CD40 agonism promotes dendritic cell (DC) maturation, enhances antigen presentation, upregulates costimulatory molecules, and facilitates cross-presentation of tumor-associated antigens^22–27^. CD40 agonists have also demonstrated synergistic potential when administered with immune checkpoint inhibitors^28,29^ and have been found to have acceptable safety profiles with evidence of clinical activity in human clinical trials^30–33^. Agonistic CD40 therapy has the potential to bridge the gap between innate and adaptive immunity by enabling robust CD8 + T cell priming and activation, potentially overcoming the immunosuppressive barriers characteristic of ER + breast cancers. Our present study evaluates the ability of aCD40 to improve anti-tumor immunity via DC activation and subsequent T cell priming and activation and to synergize with checkpoint blockade in a syngeneic mouse model of ER + breast cancer. Our findings suggest agonistic CD40 therapy may be a viable strategy for immune activation and long-lasting immunity in ER + breast cancer.

## Results

### aCD40 enhances ERα + mammary tumor sensitivity to immune checkpoint blockade

**Figure 1:**
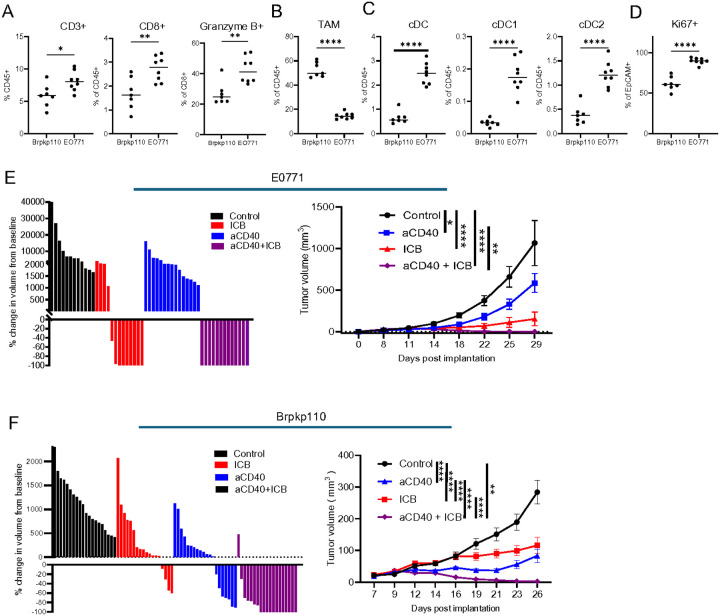
aCD40 increases ERα+ mammary tumor sensitivity to ICB. A-C. Proportions of indicated cell types in the TME of Brpkp110 and E0771 tumors by flow cytometry on day 13 post implantation (n=7–8). D. Proportions of Ki67+ proliferating EpCAM+ tumor cells in vivo measured by flow cytometry on day 13 post implantation (n=7–8). E. Tumor volume changes compared to pretreatment on day 29 post implantation (left) and growth curves (right) of E0771 tumor cells. Indicated treatments initiated on day 8 post implantation (n=12–15, data representative of 2 experiments with similar results). F. Tumor volume changes compared to pretreatment on day 26 post implantation (left) and growth curves (right) of Brpkp110 tumor cells. Indicated treatments initiated on day 7 post implantation (n=18–20, data representative of 3 experiments with similar results). Data: (A-D) median, (E-F, left) each column represents individual tumor, (E-F, right) mean ± SEM. For all panels, p<0.05 was considered statistically significant, and * p<0.05, ** p<0.01, ***p<0.001 and ****p<0.0001.

TNBC and ERα + breast cancer differ clinically in their response to chemotherapy and immunotherapy, with hormone receptor-positive cancers being more resistant to ICB^11,12^. It is well established that tumor mutational burden and T-cell infiltration can predict the response to ICB treatment, with preliminary evidence suggesting this is also relevant in breast cancer^13–15,37^. To understand the potential underlying resistance mechanisms of ERα + tumors to ICB, we aimed to compare the mutational burden and tumor immune environment of ERα + Brpkp110 and TNBC E0771 mammary tumor cell lines. *In vivo*, Brpkp110 tumors express ERα by flow cytometry (Suppl. Figure 1A) and IHC (Suppl. Figure 1B left) and are responsive to estradiol supplementation (Suppl. Figure 1C). As previously described^35^, E0771 tumors lack ERα expression *in vivo* (Suppl. Figure 1B right). Next, using an in-house developed algorithm called antigen.garnish^36^, we determined the number of single nucleotide and complex variants and predicted MHCI and MHCII neoantigens for both tumor cell lines against wild-type C57BL/6J. The two tumor cell lines displayed comparable levels of variants and predicted neoantigens (Suppl. Figure 1D), but exhibited differences in the tumor immune environment when implanted orthotopically into the fat pads of congenic mice. Consistent with human breast cancer, ERα + Brpkp110 tumors are more immunologically “cold” compared to triple-negative E0771 tumors, with decreased intratumoral T cell infiltration and increased frequency of tumor-associated macrophages (TAM) (Fig. 1A–B). Both the abundance of CD8 + T cells and the proportion of Granzyme B + CD8 + cytotoxic T cells (CTL) were lower in the ERα + Brpkp110 tumors compared to TNBC. ERα + Brpkp110 tumors also had significantly decreased numbers of type 1 and type 2 conventional dendritic cells (cDC1 and cDC2) suggesting a weakened capacity for T cell priming considered critical for anti-tumor immunity (Fig. 1C). Although TNBC E0771 tumors had a more highly activated immune environment, the tumors continued to demonstrate more aggressive tumor characteristics, including higher proportions of Ki67 + proliferating EpCam + tumor cells (Fig. 1D). As expected, when implanted orthotopically, untreated E0771 grew faster acquiring almost 3-fold larger volumes within the same period as ERα + Brpkp110 tumors (Fig. 1E–F). Upon treatment with ICB (αCTLA-4 and αPD-1), 70% of E0771 tumors regressed, and 53% of all treated mice were cured by day 29 post-implantation (Fig. 1E). In contrast, only 22% of ICB-treated Brpkp110 tumors regressed, and none of the treated hosts were cured (Fig. 1F), recapitulating the ICB-resistant phenotype of ERα + human tumors. The very low cDC content in ERα + tumors may result in poor T cell priming, activation, and recruitment, rendering ICB treatment an insufficient mechanism for reversal of tumor-associated immunosuppression. When treated with aCD40, the growth of Brpkp110 tumors was suppressed across the group with 37% tumor regressions. In contrast, the response of E0771 tumors to aCD40 was very modest, with no regressions. However, combining aCD40 and ICB cured 100% of E0771 and 63% of Brpkp110 tumor-bearing mice. Collectively, these data demonstrate that Brpkp110 can be utilized as a model to study the immunobiology of ERα + breast cancer and that aCD40 can overcome the obstacles underlying the ERα + tumor resistance to ICB.

### aCD40 increases tumor-infiltrating cytotoxic T-cells

**Figure 2:**
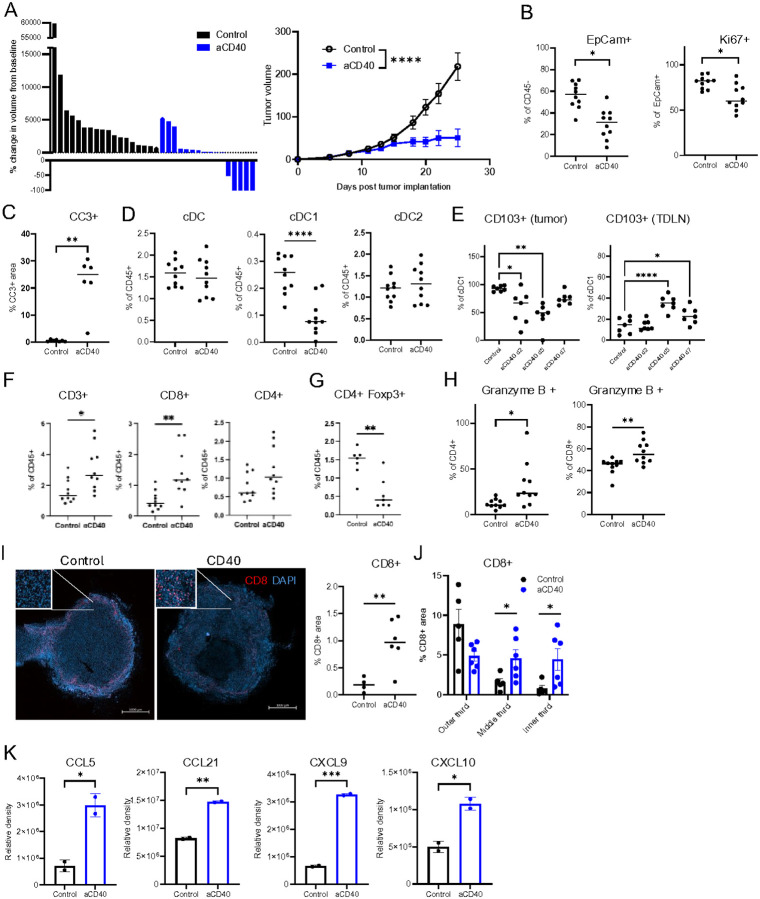
aCD40 increases tumor-infiltrating cytotoxic T-cells in orthotopic ERα+ Brpkp110 tumors. A. Tumor volume changes compared to pretreatment on day 25 post implantation (left) and growth curves (right) of Brpkp110 tumor cells. aCD40 or vehicle treatments initiated on day 6 post implantation (n=16–18, data representative of 3 experiments with similar results). B. Flow cytometry analysis of implanted control and aCD40 treated Brpkp110 tumors on day 7 post treatment (n=10, data representative of 2 experiments with similar results). C. Cleaved caspase 3 measured by IHC in control and aCD40 treated Brpkp110 tumors on day 5 posttreatment (n=6–7, data representative of 2 experiments with similar results). D. DC subtype proportions measured by flow cytometry in implanted control and aCD40 treated Brpkp110 tumors on day 7 post-treatment (n=10, data representative of 2 experiments with similar results). E. Proportions of CD103+ cDC1s measured in Brpkp110 tumors (left) and TDLN (right) on days 2, 5, and 7 post aCD40 administration, compared to untreated (control) tumors (n=6–7). F. Proportions of CD3+, CD8+, and CD4+ T cells by flow cytometry in control and aCD40 treated Brpkp110 tumors on day 13 post treatment (n=10, data representative of 2 experiments with similar results). G. FoxP3+ CD4+ regulatory T cells on day 7 post-treatment (n=7, data representative of 2 experiments with similar results). H. Proportions of Granzyme B+ T cells in subpopulations by flow cytometry in control and aCD40 treated Brpkp110 tumors on day 7 post implantation (n=10, data representative of 2 experiments with similar results). I. Images of immunofluorescent staining for CD8 (red) and nuclei (blue) and CD8 staining quantification in untreated (control) and aCD40 treated Brpkp110 tumors on day 7 post treatment (n=4–6). J. Quantification of CD8 immunofluorescent staining in outer, middle, and inner thirds of control and aCD40 treated tumors on day 7 post treatment (n=5–6). K. After 7 days of treatment with aCD40, Brpkp110 tumors were minced and cultured ex vivo. Supernatant was collected and pooled for each treatment group after 48 hours and cytokines were measured. (n=2, with 3 tumors pooled per group). Data: (A, left) each column represents individual tumor and (A, right) mean±SEM, (B-G, H right, K) median, (I, J) mean±SEM. * p<0.05, ** p<0.01, *** p< 0.001, and ****p<0.0001.

To understand how aCD40 changes the TME and makes ERα + tumors more responsive to ICB, the Brpkp110 tumor-bearing mice were treated with aCD40 only. Interestingly, aCD40 alone cured 25% of treated mice (Fig. 2A). The TME analysis 7 days after a single dose of aCD40 revealed fewer EpCam + tumor cells and a lower proportion of Ki67 + proliferating tumor cells in treated tumors (Fig. 2B). Tumor cell apoptosis was also increased in treated tumors as evidenced by increased staining for cleaved caspase 3 (Fig. 2C). These changes in tumor cell population are unlikely to be a direct effect of aCD40 on tumor cells as only 1% of them expressed CD40 *in vitro* (Suppl. Figure 2A). In contrast about one-third of total cDCs, cDC1s, and cDC2s in untreated Brpkp110 tumors were CD40+ (Suppl. Figure 2B). Interestingly, there were no changes in total cDC and cDC2 populations between control and treated groups but there was a significant decrease in cDC1 population on treatment day 7 (Fig. 2D). The time course of the changes in CD103 + migratory subpopulation of cDC1s showed that already on day 2 and 5 of aCD40 treatment, there was a significant decrease in the proportion of these cells in the tumor with a concomitant increase of the same cell population in tumor draining lymph node (TDLN) on days 5 and 7 (Fig. 2E). On day 13 post-treatment, the treated tumors had a higher percentage of CD3 + and CD8 + T cells and a trend towards a higher percentage of CD4 + T cells compared to the controls (Fig. 2F). In addition to an increase in activated CD8 + T cells, aCD40 treated tumors demonstrated decreased proportions of immunosuppressive CD4 + Foxp3 + regulatory T cells (Fig. 2G). CD4 + and CD8 + T cells appeared more activated in aCD40 treated tumors as the proportion of Granzyme B + cells was higher in these populations (Fig. 2H). We confirmed the increase in CD8 + T cells in treated tumors by IF staining (Fig. 2I). In addition to increasing the number of tumor-infiltrating CD8 + T cells, aCD40 prompted their translocation from the tumor periphery to the center of the tumor (Fig. 2J). The increase in T cells in the TME after aCD40 treatment was possibly due to the increase in the production of chemokines such as CCL5, CCL21, CXCL9, and CXCL10 within the tumor (Fig. 2K). Thus, aCD40 treatment reshaped the TME of ERα + Brpkp110 tumors by prompting DC migration to TDLN and subsequent T cell priming, activation, and recruitment into the tumor.

### cDC1s and T cells are critical for the anti-tumor activity of aCD40

**Figure 3:**
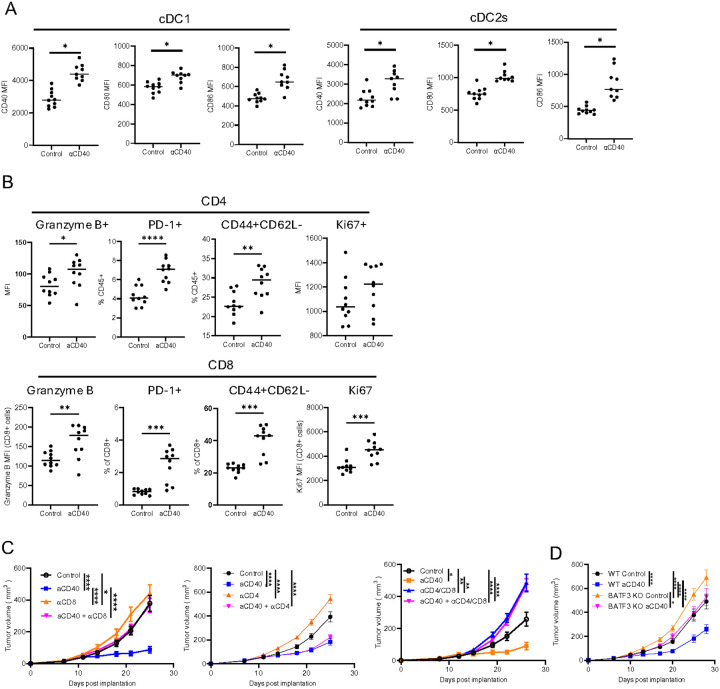
cDC1s and T cells are critical for the anti-tumor activity of aCD40. A. DC maturation and activation markers measured by flow cytometry in cDC1 and cDC2 cell populations of control and aCD40 treated Brpkp110 TDLN on day 2 post-treatment (n=8–10, data representative of 2 experiments with similar results). B. T cell activation and proliferation markers measured by flow cytometry in CD4+ and CD8+ T cell populations of control and aCD40 treated Brpkp110 TDLN on day 7 post-treatment (n=10, data representative of 2 experiments with similar results). C. Growth curves of control and aCD40 treated Brpkp110 tumors implanted into WT hosts with or without T cell depletions (n=17–20). D. Growth curves of control and aCD40 treated Brpkp110 tumors implanted into WT and BATF3 KO hosts (n=16–20). Data: (A-B) median, (C-D) mean±SEM. * p<0.05, ** p<0.01, *** p< 0.001, and ****p<0.0001.

aCD40 treatment induced maturation and activation of cDC1s and cDC2s in the lymph nodes, with elevated expression of CD40, CD80, and CD86 in TDLN infiltrating cDCs two days after aCD40 administration (Fig. 3A). At the same time point, CD4 + and CD8 + cells in TDLN appeared to be more activated after aCD40 treatment and exhibited higher Granzyme B expression and higher proportions of PD-1 and CD44 expressing cells (Fig. 3B). In addition, cell proliferation as measured by Ki67 was increased in CD4 + and CD8 + T cells after aCD40 treatment (Fig. 3B). To assess the dependency on T cells for the therapeutic effect of aCD40 in ERα + tumors, tumor-bearing control and treated mice were depleted of CD8 + or CD4 + or both CD4 + and CD8 + T cells (Suppl. Figure 3A). The tumor suppressive effect of aCD40 was abolished in the absence of CD8 + or CD8 + and CD4 + T cells, but was unaffected in hosts that were depleted of only CD4 + T cells (Fig. 3C). Similarly, there was a significant impact on tumor growth suppression after aCD40 when tumors were implanted into Batf3 knock out (KO) mice that lack cross-presenting cDC1s (Suppl. Figure 3B and Fig. 3D). These data indicate that aCD40 tumor-suppressive effect heavily relies on the presence and function of cDC1 and CD8 + cytotoxic T cells (CTL) and may be able to substitute for CD4 + help.

#### Combination aCD40 and ICB treatment results in a vaccine effect and allows for subsequent rejection of rechallenge tumors

**Figure 4:**
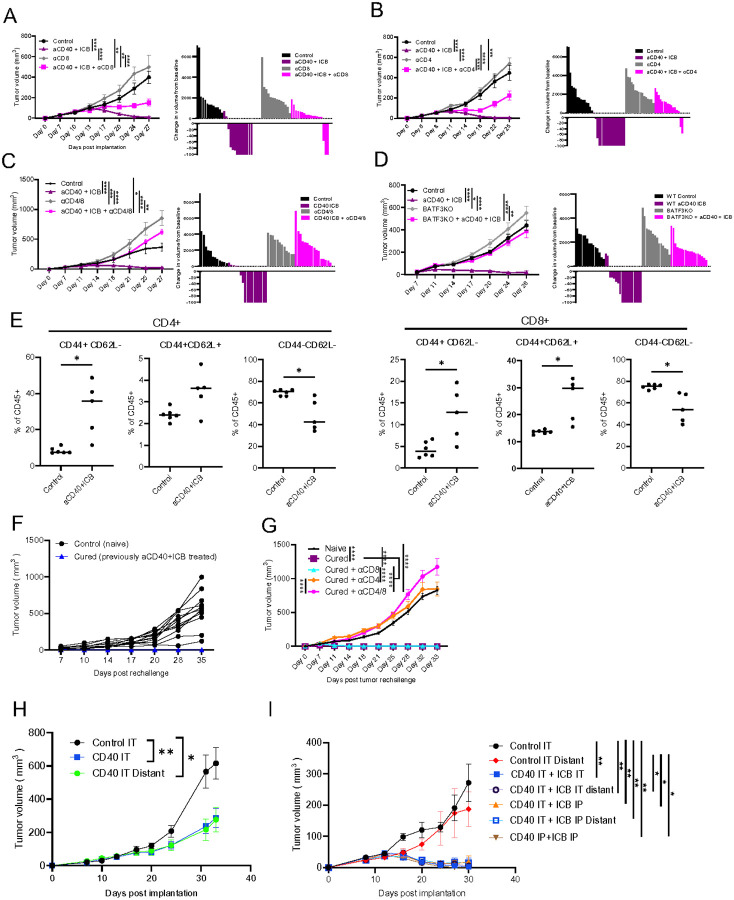
aCD40 and ICB results in a vaccine effect and rejection of a secondary tumor rechallenge. A. Brpkp110 tumor growth curves (left) and volume changes compared to pretreatment (right) on day 27 post implantation in control and aCD40+ICB treated hosts with and without CD8+ T cell depletions. Indicated treatments initiated on day 7 post implantation (n=12–15). B. Brpkp110 tumor growth curves (left) and volume changes compared to pretreatment (right) on day 25 post implantation in control and aCD40+ICB treated hosts with and without CD4+ T cell depletions. Indicated treatments initiated on day 8 post implantation (n=14–18). C. Brpkp110 tumor growth curves (left) and volume changes compared to pretreatment (right) on day 27 post implantation in control and aCD40+ICB treated hosts with and without CD4+ and CD8+ T cell depletions. Indicated treatments initiated on day 7 post implantation (n=12–18). D. Tumor growth curves (left) and volume changes compared to pretreatment (right) on day 26 post Brpkp110 tumor implantation into WT and BATF3 KO hosts. Indicated treatments initiated on day 7 post implantation (n=14–18). E. Proportions of circulating effector memory (CD44+CD62L−), central memory (CD44+CD62L+), and naïve (CD44−CD62L−) CD4+ (left) and CD8+ (right) in blood, 3 months post treatment induced tumor clearance (n=5–6, data representative of 2 experiments with similar results). F. Secondary Brpkp110 tumor rechallenge of naïve and previously Brpkp110 tumor bearing mice cured after aCD40 + ICB, at least 2 months post primary tumor clearance (n=12–14, data representative of 3 experiments with similar results). G. Control and rechallenge tumor growth in T cell sufficient (n=6–12) and T cell depleted hosts (n=12–14, data representative of 2 experiments with similar results). H. Brpkp110 tumor growth curves in intra-tumoral (IT) vehicle (control) and IT aCD40 treated hosts. aCD40 administered tumors denoted as aCD40 IT and contralateral untreated tumors denoted as CD40 IT Distant (n=9–12, data representative of 2 experiments with similar results). I. Brpkp110 tumor growth curves in intra-tumoral (IT) vehicle (control) and IT or intraperitoneal (IP) aCD40 or ICB received hosts (n=4–9, data representative of 2 experiments with similar results). * p<0.05, ** p<0.01, *** p< 0.001, and ****p<0.0001.

The influx of T cells into the TME and their activation after aCD40 treatment inevitably leads to T cell exhaustion, potentially increasing sensitivity to ICB. aCD40 alone suppressed the growth of ERα + tumors; however, only one-fourth of tumors had complete regressions (Fig. 2A), while the combination of aCD40 and ICB increased the complete response rate up to 83% (Fig. 1F, Fig. 4A–D). Interestingly, the effect of the combination therapy was only partially reversed by depletion of only CD8 + or CD4 + T cells (Fig. 4A and B, respectively), whereas combined CD8 + and CD4 + T cell depletion completely abolished the effect of aCD40 + ICB treatment (Fig. 4C). Tumors implanted in Batf3 KO hosts were resistant to combination therapy (Fig. 4D). The effect of combination immunotherapy on T cells persisted beyond the onset of tumor clearance. Two months after complete tumor regressions and cessation of treatment, the proportion of CD44 + CD62L− effector memory and CD44 + CD62L + central memory CD4 + and CD8 + T cells were higher in the blood of cured mice compared to treatment-naïve mice implanted with tumors (Fig. 4E). At the same time, cured mice had fewer circulating CD44−CD62L− naïve T cells compared to treatment-naïve mice (Fig. 4E). The presence of effector and central memory T cells in cured mice is consistent with a vaccine effect and continued anti-tumor immunosurveillance and immunologic memory. To test the robustness of this immunologic memory, cured mice were rechallenged with the same burden of ERα + Brpkp110 tumor cells. The cells grew in control, tumor naïve mice but were universally rejected in cured mice (Fig. 4F). The rejection of tumors upon secondary rechallenge was only CD4 + T cell-dependent and not CD8 + T cell dependent, as the cured hosts depleted of only CD8 + T cells were able to reject a secondary tumor rechallenge similar to the cured, T cell sufficient hosts (Fig. 4G).

### Intratumoral administration of aCD40 + ICB suppresses the growth of treated and distant tumors

Intratumoral (IT) administration of ICB and aCD40 have individually been shown to be efficacious in human clinical trials without significant systemic absorption^38–40^. Here we tested the efficacy of IT aCD40 alone or in combination with IT ICB in double-flanked Brpkp110 tumor-bearing mice. IT aCD40 suppressed the growth of both the treated ipsilateral tumor as well as the distant contralateral tumor (Fig. 4H). Next, various combinations of IT and intraperitoneal (IP) administrations of aCD40 and ICB were tested in double-flanked hosts. IT aCD40 and ICB were able to suppress both the treated and distant tumors, similar to the effect observed in IP treatments (Fig. 4I), indicating that local administration of aCD40 + ICB results in an abscopal effect on distant tumors.

## Discussion

In the current study, we sought to elucidate the mechanisms underlying ER + tumor resistance to ICB by comparing immunological profiles of ER + Brpkp110 and TNBC E0771 murine mammary tumors. Differences were observed in their immune infiltration profiles despite similar mutational burdens and neoantigen loads between these tumor models. TNBC E0771 tumors exhibited a T cell-inflamed, “hot” TME characterized by robust CD8 + T cell infiltration and higher proportions of Granzyme B + cytotoxic T lymphocytes (CTLs), alongside greater densities of dendritic cells. In contrast, ER + Brpkp110 tumors displayed an immunologically “cold” TME with minimal lymphocytic infiltration. These distinctions in immune contexture, rather than mutational burden, correlated with differential ICB responsiveness.

These findings suggest that immune resistance in ER + tumors stems at least in part from deficient T cell priming, activation, and recruitment, rather than acquired dysfunction of activated T cells within the TME. This hypothesis provided the rationale for targeting the CD40 pathway in order to enhance dendritic cell maturation and antigen presentation^19,23^. In various immunologically “cold” tumor models, including pancreatic adenocarcinoma and glioblastoma, agonistic CD40 antibodies (aCD40) have successfully activated cDC1s, improved T cell priming, and enhanced anti-tumor immunity^21,41^. Consistent with our mechanistic hypothesis, we found that aCD40 monotherapy exhibited robust tumor-suppressive effects in ER + tumors compared to TNBC. The combination of aCD40 and ICB (αPD1 and αCTLA-4), addressing both pre- and post-T cell activation defects, resulted in complete tumor clearance in the majority of TNBC and ER + tumor-bearing mice, representing unprecedented efficacy in an ER + murine breast cancer model.

Our temporal analysis of aCD40 effects revealed a sequential cascade of immunological events. Following a single dose of aCD40, we observed a significant reduction in intratumoral CD103 + migratory DCs by days 5–7 post-treatment, concurrent with increased accumulation of mature, activated cDC1 and cDC2 in tumor-draining lymph nodes (TDLNs), accompanied by robust proliferation and activation of CD4 + and CD8 + T cells. These findings parallel prior observations in pancreatic cancer models^22^ and highlight aCD40’s ability to increase DC mobilization into the TDLN.

The immunomodulatory effects of aCD40 also result in the induction of a pro-inflammatory chemokine milieu. aCD40 treatment significantly increased tumoral expression of CCL5, potentially contributing to the recruitment of Granzyme B + CD4 + T cells from TDLNs to the tumor bed, as previously reported in pancreatic cancer models^42^. Concomitantly, we observed a marked reduction in Foxp3 + regulatory T cells within treated tumors, suggesting a shift in the CD4 + T cell population from an immunosuppressive to an immunostimulatory phenotype. Additionally, aCD40 enhanced tumoral production of CXCL9 and CXCL10, potent T cell chemoattractants that likely facilitated T cell trafficking from the periphery to the tumor microenvironment.

An intriguing observation was the aCD40-induced upregulation of CCL21 within treated tumors. CCL21, traditionally produced by lymphatic endothelial cells and lymph node stromal cells, acts as a chemotactic signal for CCR7-expressing DCs to traffic to lymph nodes^43^. While CD103 + CCR7 + cDC1s represent the canonical antigen-trafficking migratory DCs responsible for CD8 + T cell priming^44^, recent evidence indicates that a subset of CCR7 + immature DCs that downregulate antigen presentation machinery and upregulate PD-L1 can undermine T cell priming in lymph nodes^45^. The increased CCL21 production within aCD40-treated tumors may serve to retain these regulatory DCs within the tumor microenvironment, thereby facilitating more effective T cell priming in TDLNs. Simultaneously, the presence of PD-L1-expressing DCs within tumors could impair T cell effector function, potentially explaining why ICB complements aCD40 in achieving maximal therapeutic efficacy. However, further investigation is warranted to fully elucidate the functional significance of enhanced CCL21 expression in this context.

Through selective depletion studies, we established that initial therapeutic tumor regression following aCD40 monotherapy critically depends on CD8 + T cells but not CD4 + T cells, similar to prior data demonstrating that aCD40 can substitute for CD4 + T cell help^46^. The absence of Batf3-dependent cross-presenting cDC1s also abolished a significant proportion of the aCD40 anti-tumor effect, underscoring the essential role of cDC1s in initiating CD8 + T cell-mediated tumor immunity. In contrast, the efficacy of combined aCD40 and ICB treatment required both CD4 + and CD8 + T cells along with cDC1s for primary tumor rejection, highlighting the cooperative nature of these immune populations in mediating maximal therapeutic responses in the context of combination immunotherapy. The durable nature of the anti-tumor immunity was evidenced by elevated proportions of circulating effector and central memory T cells two months after tumor clearance, as well as the ability of cured mice to reject secondary tumor rechallenge, confirming the establishment of immunological memory via a vaccine effect.

In contrast, while primary tumor rejection required both CD4 + and CD8 + T cells, rechallenge tumor clearance demonstrated dependency on CD4 + T cells alone. This unexpected finding suggests that in the context of established immune memory, CD4 + T cells may either acquire direct cytotoxic functionality or orchestrate anti-tumor responses through cell populations other than CD8 + T cells. This observation warrants further investigation to delineate the precise mechanisms by which CD4 + T cells mediate tumor rejection in the memory phase.

Finally, our demonstration that intratumoral administration of aCD40, either alone or in combination with ICB, recapitulates the effects of systemic treatment has significant clinical implications. This finding indicates that localized immunomodulation within the primary tumor and associated TDLNs is sufficient to induce regression of distant lesions, potentially through the systemic dissemination of tumor-specific T cells. This approach could mitigate immune-related adverse events associated with systemic administration of immunotherapeutics, while maintaining therapeutic efficacy. Moreover, our data suggest that TDLNs play a crucial role in controlling distant metastatic lesions and preventing recurrence, arguing for the potential conservation of TDLNs during surgical resection to maximize the endogenous vaccine effect of the primary tumor.

In summary, we demonstrate for the first time complete reversal of ICB resistance in ER + breast cancer through CD40 agonism. By converting immunologically “cold” ER + tumors into responsive entities through the activation of dendritic cells and enhancement of T cell priming, our findings provide a strong rationale for clinical investigation of combined aCD40 and ICB in ER + breast cancers. This approach may dramatically expand the proportion of breast cancer patients who can benefit from immunotherapy, potentially addressing a critical unmet need in the management of the most common breast cancer subtype. Furthermore, the mechanistic insights gleaned from this study may inform combination immunotherapy strategies across other immunologically “cold” tumor types characterized by deficient T cell priming and recruitment.

## Methods

### Cell lines

Brpkp110 is an estrogen receptor positive (ER+), progesterone receptor positive (PR+) and human epidermal growth receptor 2 negative (HER2−) mammary cell line derived from a tumor induced in the mammary gland of a *KRas*^*G12D−LSL/wt*^ *;p53*^*flx/flx*^ *;myr-p110*^*wt/fl*^ mouse (gift from Jose Conejo Garcia, Duke University) which leads to constitutively activated PI3K signaling^34^. Brpkp110 cells were cultured in RPMI 1640 medium containing 10% fetal bovine serum, 1% penicillin/streptomycin, 0.05% sodium pyruvate, and 3.3uL 2-mercaptoethanol. E0771 is a widely-used ERα- cell line^35^ and was cultured in DMEM, 10% FBS, 1% glutamine, and 0.2% gentamicin.

### Mice and treatments

Age- and sex-matched 7- to 18-week-old C57BL/6 mice (Jackson Laboratory, cat# 00664) and B6.129S(C)-*Batf3*^*tm1Kmm*^/J (Jackson Laboratory, cat# 013755) were housed in specific pathogen-free conditions and treated as per an approved Institutional Animal Care and Use Committee protocol at the University of Pennsylvania (Protocol #804666). Tumors in C57BL/6 and B6.129S(C)-*Batf3*^*tm1Kmm*^/J mice were established orthotopically in paired bilateral abdominal mammary glands 9 and 4 via injection of 5e5 cells of Brpkp110 or E0771 cell lines. The treatment antibodies listed below were obtained from Bio X Cell unless otherwise indicated. Tumor-bearing C57BL/6 or B6.129S(C)-*Batf3*^*tm1Kmm*^/J mice received one dose of an agonistic CD40 antibody (clone FGK4.5, 100 μg intraperitoneal (i.p.) or intratumoral (i.t.)) or rat IgG2a (clone 2A3, 100 μg i.p. or i.t.) on day 0 as previously described^28^. Anti-CTLA-4 (clone 9H10, 200 μg i.p. or i.t.) or rat IgG2b (clone LTF-2, 200 μg i.p. or i.t.) were given on day 0, 4, 7^28^. Anti-PD-1 (clone RMPI-14, 200 μg i.p. or i.t.) or rat IgG2b (clone LTF-2, 200 μg i.p. or i.t.) were given on day 0 and every 3 to 4 days thereafter^28^. Anti-CD4 (clone GK1.5, 200 μg i.p.) or rat IgG2b (clone LTF-2, 200 μg i.p.) was given on day − 4, day 0, and every 3 to 4 days thereafter for CD4 T-cell depletion. Anti-CD8 (clone 2.43, 200 μg i.p.) or rat IgG2b (clone LTF-2, 200 μg i.p.) was given on day − 4, day 0, and every 3 to 4 days thereafter for CD8 T-cell depletion.

### Tissue processing and flow cytometry

Mice were sacrificed according to IACUC approved protocols and tumors and tumor-draining lymph nodes (TDLN) were collected. Tumors were digested in 1 mg/mL collagenase with protease inhibitor (MilliporeSigma) and filtered through a 70 μm cell filter. TDLNs were mechanically dissociated and filtered using 100 μm filters. Cells were resuspended in PBS and incubated in Fc block (Biolegend 3527448) for 5 minutes on ice prior to staining for flow cytometry. The live/dead stain Zombie UV was used. Cells were stained with Zombie UV live/dead stain and for cell surface markers at 4°C in the dark for 30 minutes. Cell suspensions were then fixed and permeabilized for 30 minutes (eBioscience Intracellular Fixation & Permeabilization Buffer Set, Invitrogen) and overnight staining was performed for intracellular targets. Conjugated antibodies used for flow cytometry were obtained from BD Biosciences [PD-L1 (clone MIH5), CD4 (clones GK1.5, RM4–5), CD103 (clone M290), CD80 (clone 16–10A1), FoxP3 (clone R16–715)], Biolegend [EpCam (clone G8.8), CD45 (clone 30-F11), Ki67 (clone 16A8), Granzyme B (clone AQA16A02), CD8 (clone 53 − 6.7), CD3 (clone 145–2C11), CD40 (clone 3/23), CD86 (clone G2–1), PD-1 (clone 29F.1A12), CD19 (clone 6D5), B220 (clone RA3–6B2), CD11c (clone N418), NK1.1 (clone PK136), Gr-1 (clone RB6–8C5), XCR1 (clone ZET), CD11b (clone M1/70), CD64 (clone X54–5/7.1), SIRPα (clone P84), CD44 (clone IM7), CD62L (clone MEL-14)], and Abcam [ERα (clone E115). Flow cytometry was performed using an LSRFortessa or FACSymphony A3 Cell Analyzer and data were analyzed using FlowJo v10.8 software (BD Biosciences).

### Immunohistochemistry and immunofluorescence

Tumor tissues were fixed in Zinc formalin for 24 hours and embedded in paraffin. Immunohistochemistry (IHC) for ERα+ (clone E115, Abcam 32063, 1:9000) and cleaved caspase-3 (Cell Signaling Technology 9661, 1:500) was performed by the Comparative Pathology Core of the University of Pennsylvania School of Veterinary Medicine. Immunofluoresence (IF) for CD8 and DAPI (Thermo Scientific D21490,1:1000) was performed according to the Immunofluorescent Staining of Paraffin-embedded Tissue Protocol from Novus Biologicals, with certain modifications as follows. Antigen retrieval was performed using the IHC-Tek Epitope Retrieval Solution (IHC World 1W-1100), with slides incubated for 45 minutes in an IHC World steamer at 95–98°C. Tissue sections were blocked with 5% donkey serum diluted in PBS with 0.3% Triton X-100 and stained with rabbit anti–mouse primary CD8 (clone: D4W2Z, Cell Signaling Technology 98941, 1:100) followed by donkey anti-rabbit Alexa Fluor 594 (Thermo Scientific A-21207, 1:250) secondary antibody. Slide scanning was performed on Aperio Versa 8 slide scanner at the Molecular Pathology and Imaging Core facility at the University of Pennsylvania. Data were analyzed using QuPath. In short, QuPath’s wand function was utilized to draw a border around the DAPI-stained tissue area. Then, the “Thresholder” function was utilized to gate for positive antibody-staining, in both IF and IHC, which could be visualized in real-time. Once an appropriate staining threshold value was determined for positive-staining gating it was applied to whole tissue sections. QuPath quantified percent positive area for each antibody was used for analysis.

### Cytokine analysis

Brpkp110 tumor-bearing mice underwent treatment as described in results. Mice were euthanized and tumors were collected at day 7 post treatment. Tumors were minced and incubated in media for 48 hours. Supernatant was collected and pooled per treatment group and cytokine array was performed with technical duplicates via the proteome profiler (R&D, catalog # ARY028).

### Whole exome sequencing

Genomic DNA was extracted from Brpkp110 and E0771 cell pellets and livers of wild-type C57BL/6 mice. Whole exome sequencing was performed on the Illumina platform (2×150bp) by Azenta Life Sciences. Neoantigen prediction was performed via antigen.garnish^36^. Briefly, we applied antigen garnish to identify single nucleotide variants (SNVs) and SNV-induced protein alterations and ran mutated peptides (8mers to 14mers) through a series of MHC-binding affinity platforms (netMHCI/netMHCII/netMHCIpan/netMHCIIpan). Identified peptides with predicted binding affinities less than 500 nM were prioritized as strong binders.

### Statistical analysis

Tumor growth curves were analyzed using two-way ANOVA with Tukey multiple comparisons of means to compare differences between two individual groups. A two-tailed Student *t* test was used to analyze differences between two groups. One-way ANOVA with the Bonferonni multiple comparison test was used to assess differences between any two individual groups. Statistical analyses were performed using GraphPad Prism 10. *P* ≤ 0.05 was considered statistically significant.

## Supplementary Files

This is a list of supplementary files associated with this preprint. Click to download.

- 060425FiguresforNPJBCSupp.pdf

## Data Availability

The datasets generated and/or analyzed during the current study are available in the Sequence Read Archive (SRA) repository accession code SUB15367120.
